# A plant Bcl-2-associated athanogene is proteolytically activated to confer fungal resistance

**DOI:** 10.15698/mic2016.05.501

**Published:** 2016-04-16

**Authors:** Mehdi Kabbage, Ryan Kessens, Martin B. Dickman

**Affiliations:** 1 University of Wisconsin-Madison, Department of Plant Pathology, Madison, WI 53706.; 2 Texas A&M University, Department of Plant Pathology and Microbiology, Institute for Plant Genomics and Biotechnology, College Station, TX 77843.

**Keywords:** BAG, Aspartyl Protease, Autophagy, Botrytis cinerea, Fungal Resistance, Basal Immunity

## Abstract

The Bcl-2-associated athanogene (BAG) family is a multifunctional group of proteins involved in numerous cellular functions ranging from apoptosis to tumorigenesis. These proteins are evolutionarily conserved and encode a characteristic region known as the BAG domain. BAGs function as adapter proteins forming complexes with signaling molecules and molecular chaperones. In humans, a role for BAG proteins has been suggested in tumor growth, HIV infection, and neurodegenerative diseases; as a result, the BAGs are attractive targets for therapeutic interventions, and their expression in cells may serve as a predictive tool for disease development. The *Arabidopsis* genome contains seven homologs of BAG family proteins (Figure 1), including four with a domain organization similar to animal BAGs (BAG1-4). The remaining three members (BAG5-7) contain a predicted calmodulin-binding motif near the BAG domain, a feature unique to plant BAG proteins that possibly reflects divergent mechanisms associated with plant-specific functions. As reported for animal BAGs, plant BAGs also regulate several stress and developmental processes (Figure 2). The recent article by Li *et al.* focuses on the role of BAG6 in plant innate immunity. This study shows that BAG6 plays a key role in basal plant defense against fungal pathogens. Importantly, this work further shows that BAG6 is proteolytically activated to induce autophagic cell death and resistance in plants. This finding underscores the importance of proteases in the execution of plant cell death, yet little is known about proteases and their substrates in plants.

While BAG-like genes appear to be widely distributed in plants, they have not been characterized to the same extent as in mammals, and functional studies are limited. In plants, BAG6 was first identified in a screen for calmodulin binding proteins, and later was shown to be essential for basal resistance against the plant necrotrophic fungal pathogen *Botrytis cinerea*. The work by Li *et al.* focuses on the molecular mechanisms that underpin BAG6-mediated resistance to this pathogen. Initial bioinformatic analysis revealed that BAG6 contains a potential capase-1 cleavage site (LATD) downstream of its BAG domain. Purification of BAG6 and treatment with caspase-1 resulted in cleavage of wild type BAG6 but not the BAG6D760A mutant, in which aspartate (D) was mutated to alanine (A) in the caspase-1 cleavage site. These results show that this caspase-1 recognition site is operational and may be relevant to BAG6 function. It is important to note that, while plant genomes do not encode obvious homologs of animal caspases, they do encode proteases with caspase-like activity. Vacuolar processing enzymes (VPEs), subtilisin-like serine proteases (subtilases), and aspartic proteases are all known to cleave after aspartate and recognize similar cleavage sites as caspases.

**Figure 1:**
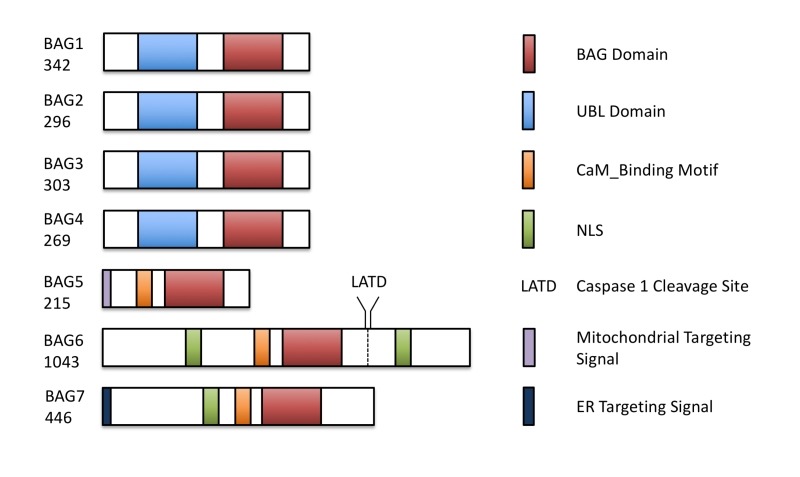
FIGURE 1: The structures of the Arabidopsis BAG-family proteins are illustrated, including the conserved BAG domain, ubiquitin-like (UBL) domain, nuclear localization signal (NLS), and Calmodulin-binding motif (CaM). BAG5 and BAG7 contain mitochondrial and ER targeting peptides, respectively. A functional caspase-1 cleavage site is present downstream of the BAG domain in BAG6. Protein length in amino acids is displayed under each BAG.

The observation that cleavage of BAG6 was triggered in response to *B. cinerea* challenge and chitin treatment suggested that BAG6 processing plays a functional role in plant defense responses to *B. cinerea*. In support of this hypothesis, *Arabidopsis* expressing *BAG6^D760A^* in the *bag6* mutant background were highly susceptible to *B. cinerea* infection. In an effort to identify proteins required for BAG6 cleavage, a pull-down assay followed by mass spectrometry was performed to detect BAG6 binding partners *in planta*. Mass spectrometric analysis of a protein band pulled down by BAG6 identified a sequence homologous to a C2- and GRAM-domain- containing protein that was designated BAGP1. Unfortunately, this method failed to deliver any proteases that could be responsible for BAG6 cleavage. To narrow the search for plant proteases responsible for BAG6 cleavage, a suppressor screen was performed using protease inhibitors that could abolish BAG6 cleavage in response to chitin treatment. Only pepstatin, an aspartyl protease inhibitor, prevented BAG6 cleavage upon chitin treatment. A computational analysis to detect known and predicted protein interactions, suggested that an aspartyl protease (designated APCB1) may be a BAGP1-interactor. To confirm this interaction, yeast two-hybrid and pull-down assays were employed to show that all three proteins (BAG6, BAGP1, and APCB1) interacted, possibly in a complex. Furthermore, BAGP1 and APCB1 were confirmed to be essential for BAG6-mediated resistance against *B. cinerea*. *Arabidopsis bagp1* and *apcb1* T-DNA insertion lines were obtained and were equally susceptible to *B. cinerea* infection as the *bag6* mutant line. Additionally, cleavage of BAG6 was markedly reduced in both *bagp1* and *apcb1* mutants. 

Previous studies in plants and animals have attributed a role for BAG proteins in the regulation of autophagy. Activation of autophagy has also been shown to be an important resistance response used by plants against necrotrophic fungal pathogens. While the presence of autophagic vacuoles is clearly present in wild-type *Arabidopsis* challenged with *B. cinerea*, the *bag6* mutant lacked autophagy hallmarks. Transmission electron micrographs of tobacco cells expressing the cleaved BAG6 fragment, but not full-length BAG6, induced autophagy in the absence of pathogen challenge. To determine if the absence of autophagy in the *bag6* mutant was due to a defective autophagic machinery, *bag6* mutants were pretreated with chemical activators of autophagy. The results showed that autophagy could be induced by tunicamycin and trehalose in the *bag6* mutant, and treatment with these autophagy inducers restored resistance to *B. cinerea* in the *bag6* mutant background. Taken together, these results indicate that autophagy can still occur in the absence of BAG6 and is necessary for resistance against this pathogen.

**Figure 2:**
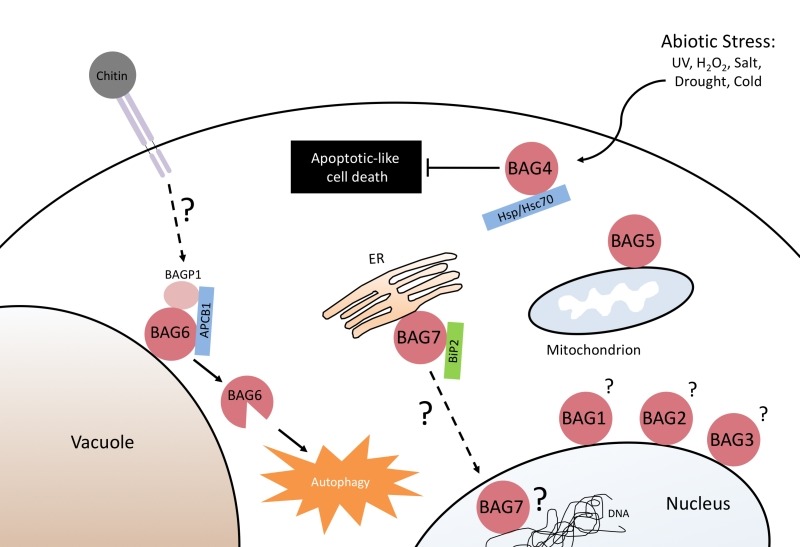
FIGURE 2: The implication of the Arabidopsis BAG-family proteins in various cellular processes linked to abiotic and biotic stress responses, and cell death regulation. The function of the nuclear BAG1-3, and mitochondrial localized BAG5 is currently unknown. BAG4 was shown to bind Hsp/Hsc 70 molecular chaperones, and is involved in cell death inhibition in response to abiotic stress. BAG6 is proteolytically activated, and functions as an adaptor protein linking fungal/chitin perception to the induction of autophagy. The ER localized BAG7, binds the molecular chaperone BiP2, and is an essential component of the unfolded protein response (UPR).

While the role of autophagy in resistance to necrotrophic pathogens is still a matter of debate, it is clear from this study and others that the activation of autophagy can often suppress disease progression. New studies are revealing that many fungal pathogens that were previously believed to lead strictly necrotrophic lifestyles do in fact have brief biotrophic phases. It is reasonable to speculate that the early and timely activation of autophagy induces localized cell death that prevents fungal establishment during its brief biotrophic phase. The study presented here provides a link between the cleavage and activation of BAG6 by an aspartyl protease and the activation of autophagy that mediates resistance against a necrotrophic fungus. Future studies should clarify the mechanism by which BAG6 activates autophagy pathways. Beyond fungal resistance, this work also informs on the key role of proteases in the execution of plant cell death regimes. The molecular details of plant programmed cell death involving proteases and their substrates is likely to provide a framework to further understand regulatory processes mediating plant cell death.

